# Marangoni effect visualized in two-dimensions Optical tweezers for gas bubbles

**DOI:** 10.1038/srep34787

**Published:** 2016-10-07

**Authors:** A. Miniewicz, S. Bartkiewicz, H. Orlikowska, K. Dradrach

**Affiliations:** 1 Advanced Materials Engineering and Modelling Group, Faculty of Chemistry, Wroclaw University of Science and Technology, 50–370 Wroclaw, Poland

## Abstract

In the report we demonstrate how, using laser light, effectively trap gas bubbles and transport them through a liquid phase to a desired destination by shifting the laser beam position. The physics underlying the effect is complex but quite general as it comes from the limited to two-dimension, well-known, Marangoni effect. The experimental microscope-based system consists of a thin layer of liquid placed between two glass plates containing a dye dissolved in a solvent and a laser light beam that is strongly absorbed by the dye. This point-like heat source locally changes surface tension of nearby liquid-air interface. Because of temperature gradients a photo-triggered Marangoni flows are induced leading to self-amplification of the effect and formation of large-scale whirls. The interface is bending toward beam position allowing formation of a gas bubble upon suitable beam steering. Using various techniques (employing luminescent particles or liquid crystals), we visualize liquid flows propelled by the tangential to interface forces. This helped us to understand the physics of the phenomenon and analyze accompanying effects leading to gas bubble trapping. The manipulation of sessile droplets moving on the glass surface induced via controlled with laser light interface bending (i.e. “droplet catapult”) is demonstrated as well.

Discovery of optical trapping of dielectric particles by focused laser beam by Ashkin *et al*.^1^,^2^,^3^,^4^ has spawned enormous interest in this phenomenon and resulted both in development of theory of light-matter interaction^5^,^6^,^7^,^8^,^9^ as well as in practical applications^10^,^11^,^12^,^13^,^14^,^15^. The possibility of motion of gas bubbles in a temperature gradient, i.e. thermocapillary effect was first observed by Young *et al*.^16^. Since that time, several groups studied the possibility of trapping and moving not only particles but also small gas or vapor bubbles in a liquid environment^17^,^18^,^19^,^20^,^21^,^22^,^23^ using other than optical gradient force mechanisms of trapping. Recent research efforts in the field of microfluidics and optofluidics is devoted to displacing, switching and trapping of bubbles and droplets using laser light as a heat source and the Marangoni effect^24^ understood as convective flow generated by the temperature gradient along a gas–liquid interface. Sophisticated use of lasers, to move and organize bubbles under various experimental conditions has been recently reported. For instance, Namura *et al*.^25^ used a focused laser beam impinging on Au nanoparticles/dielectric/Ag mirror thin film to create a micro bubble and to control the temperature gradient around it at a micrometer scale. The same group demonstrated how Marangoni flows generated around the microbubble performed focused submicron particle stream in water^26^. Oshemkov *et al*.^27^ used ultrashort laser pulses at a high repetition rate for trapping and manipulating gas bubbles in water. Hu *et al*.^28^ reported on how using optically controlled bubble microrobots a particle microassembly can be accomplished. Several papers of Matsuhara group^29^,^30^,^31^ dealt with laser induced flows and crystal formation under laser beam. Lin *et al*.^32^ reported generation of microbubbles at the interface of colloidal suspension and a plasmonic substrate via plasmon enhanced photothermal effect using single laser beam demonstrating patterned particle assemblies. Our recent reports were devoted to the physics of laser induced photonic whirls in absorbing liquids and phototropic liquid crystals^33^,^34^.

Here we demonstrate visualization of 2-D Marangoni laser induced liquid flows close to liquid-air interface and around gas bubbles. Use of fluorescent particles and special elaboration of microscopic observations enabled us precisely trace the trajectories of liquid flows. We demonstrate optical gas bubble trapping by weak cw laser beam in absorbing liquid that origins from thermocapillary Marangoni flows around the bubbles. Moreover, we demonstrate how using the thermocapillary Marangoni effect a droplet of liquid can be formed and ejected by elastic forces of gas-liquid interface.

## Results

### Physics of light-induced Marangoni effect

**Figure 1:**
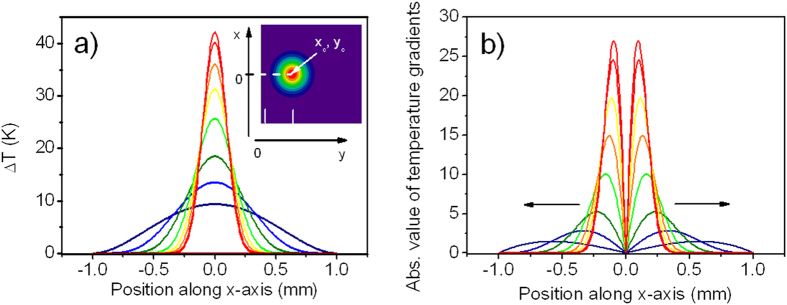
Temperature gradients due to laser heating. (**a**) The modelling of temperature distribution due to heat diffusion at different distances from the laser spot situated at (*x*_*c*_, *y*_*c*_) within an absorbing layer, color lines correspond to ΔT scale, which is exemplary but laser beam waist is real; (**b**) the absolute value of temperature gradients 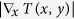 derived at the same distances from the source as in (**a**). Arrows show direction of particle movements at the interface due to temperature gradient field.

Let us consider a solution of an absorbing dye in a common organic solvent (here para-nitroaniline: C_6_H_6_N_2_O_2_ in 1,4-dioxane: C_4_H_8_O_2_)^33^. For the best results we used solutions of p-NA in 1,4-dioxane close to saturation at room temperature. The layer of a liquid is placed between two glass plates separated by distance *d* with the help of spacers so the thin film of liquid is formed in the (*x*, *y*) plane. The free surface of a liquid (|| *x*) is in contact with air at least on two opposite sides of microscope slides. The semiconductor laser beam of 70 mW power working at 405 nm is irradiating at normal incidence (along the *z*-axis) liquid layer at some distance from the interface. The beam has a Gaussian intensity profile *I*(*x*, *y*) and due to relatively strong light absorption by dissolved dye is locally heating the solution. Absorbed light energy is directly converted into heat so we can treat the beam spot as a near point-like heat source S_T_ ∼ *I*_*0*_, where *I*_*0*_ is a laser intensity at the beam center. Then the dynamic process starts. The heat released from the beam center at (*x*_*c*_, *y*_*c*_) spreads centrally by conduction heat flux *q*(*r*) and forms the distribution of a temperature according to Fourier’s law:


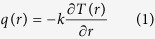


where *k* is the average thermal conductivity of the solution and the adjacent to liquid layer glass plates. When the temperature field *T*(*x*, *y*) = *T*_*a*_ + Δ*T*(*x*, *y*) arrives at the liquid-air interface situated at some distance along the *x*-axis from the point (*x*_*c*_, *y*_*c*_) a gradient of temperature appears at the interface line ∇_x_*T*(*x*, *y*), here *T*_*a*_ represents the ambient temperature. Figure 1a shows the calculated excess temperature distribution Δ*T*(*x*, *y*) at some time after beam opening around the beam center (cf. color inset to Fig. 1a) together with its sections along the *x*-direction at few chosen distances from the point (*x*_*c*_, *y*_*c*_). The heat arrives at the free interface liquid-air in the range of ms to seconds after launching laser, depending on the beam distance to the initially flat interface line. Then at the interface the temperature gradients ∇_x_*T*(*x*, *y*) arise which can be calculated taking the derivative over interface line of temperature distribution 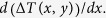 The absolute value of these gradients is shown in Fig. 1b, line colors correspond to those shown in Fig. 1a. At the x = 0 the temperature gradient is equal zero but on both sides gradients are identical in magnitude but opposite in signs having maxima diminishing in magnitude and shifting in position when interface is at larger distance from the beam center.

The line connecting laser heat source with the interface shows where the initial radial symmetry of heat transport has been broken and mirror symmetry introduced instead. The temperature distribution at the interface will change the surface tension. For most of the organic liquids, surface tension is lowering linearly with temperature increase according to the equation^35^:


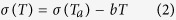


where *σ*(*T*_*a*_) is the solvent surface tension coefficient at ambient temperature T_*a*_ = 295 K, *b* = δσ/δT is the temperature coefficient typically of the order of 0.1 mN/m·K. For pure 1,4-dioxane σ(*T*_*a*_) = 33.5 mN/m and its viscosity equals to 1.177 mPa·s at 298 K and 0.787 mPa·s at 323 K. When a rise of a temperature at the interface is sufficiently large to locally decrease the surface tension by a large amount, the interfacial area with small surface tension expands at the expense of an area with greater surface tension and the important symmetric bending of the interface toward position of a heat source is observed. This bending is mainly caused by the constant air pressure *p* exerting normal force to the interface with curvature obeying the Young-Laplace equation^36^ Δ*p* = *σ*·*κ*, where κ is local curvature, i.e. *κ*(*x*, *y*) = (1/*R*_*x*_ + 1/*R*_*y*_) determined by radii *R*_*x*_ and *R*_*y*_ and Δ*p* is the pressure difference, known as the Laplace pressure. Curved surface produces a pressure that balances the external one.

It is well known that when a surface tension varies from point to point at the interface an another force with component parallel to the surface is developed that is proportional to the gradient of the surface tension^36^. This tangential force imbalance, known as Marangoni effect, results in flow of liquid directed from low to high surface tension regions *σ*(*x*, *y*). The magnitude of surface stress is given by the equation:


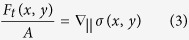


where the interfacial gradient is taken with respect to coordinates tangential to the interface.

From the temperature distribution at the interface (cf. Fig. 1a) it follows that at the closest point to a heat source (a beam spot) the gradient is zero so there is no net force contributing to flow generation. However, on both sides of the mirror line forces have identical magnitudes but opposite signs (cf. Fig. 1b). Maximum forces appear at some distance from the mirror line.

**Figure 2:**
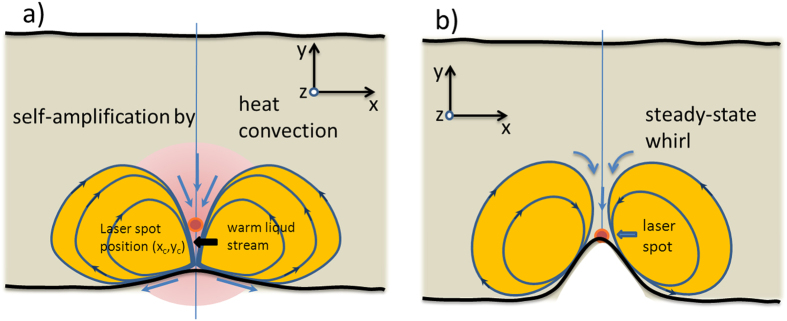
Photo-triggered Marangoni flows. Scheme of physical events for laser induced two-dimensional Marangoni effect and photonic whirl formation. (**a**) Gaussian laser beam at (*xc*, *y_c_*) incident normal to the absorbing liquid layer induces the gradient of temperature at the interface and starts the self-amplified Marangoni flows; (**b**) interface bending increases and when it reaches the laser spot position a stable whirl of fluid pinned-up to the laser spot is formed.

As a result, the two opposite in direction fluid streams build-up with the highest accelerations exactly at the points of the highest temperature gradients and close to the boundary liquid-air line. The dynamic condition for streams of liquid movement along the interface transmitting their translational movement to the adjacent liquid layers (viscous tractions) is described by the Marangoni equation expressing the balance of tangential stresses^36^:


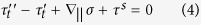


where 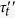 and 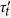 are viscous tractions of the adjacent bulk phases assuming Newtonian fluid and its respective kinematic viscosities *μ* and 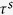 represent the contribution of any intrinsic interfacial rheology. The term ∇_||_*σ* describes Marangoni effect^24^, i.e. variation in the boundary tension caused by variation of interfacial temperature. The Marangoni effect leads to a significant bulk mass movement and heat convection toward the liquid-air interface as schematically shown in Fig. 2. Due to Bernoulli law a non-compressible liquid is flowing toward the central point at the surface from its bulk according to a stream continuity equation powered by the strength of the Marangoni effect. Initially this flow carries warmer liquid from the illuminated region of the solution toward the interface. Following heat transfer by mass displacement (heat convection) a further increase of the temperature at the liquid-air boundary takes place and is spread aside further lowering surface tension. The Nusselt number (Nu) describing the ratio of heat exchange by convection with respect to conduction rises from its initial value of 1 to higher values. The speed of mass flow can rapidly grow bringing warm liquid streams to the surface and starting from some threshold value forming loops of liquid streams powered by light. These loops are much larger (10 to 100 times) than the laser spot size and generally, they reduce the temperature gradients at the interface, so it leads to equilibration of the heat exchange process.

**Figure 3:**
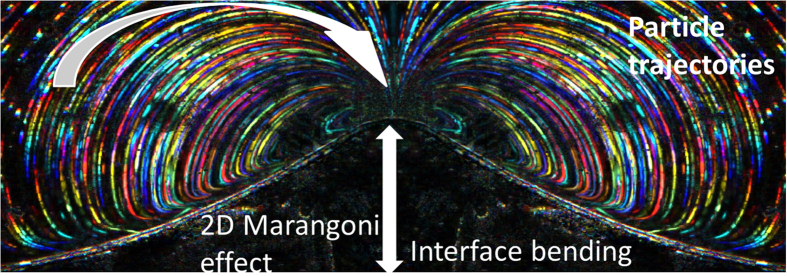
2D Marangoni effect. The composed microscopic dark field photograph of stable laser-induced whirl in pNA-1,4-dioxane solution in a thin layer. Multicolor lines represent the movement of particles in a liquid flux captured by 100 consecutive frames, each lasting 40 ms. The left side of the presented image is an inverted replica of the right side in order to underline perfect symmetry of the whirls. The lateral size of the image is about 4 mm. Vertical arrow shows interface bending amplitude due to local surface tension decrease caused by temperature distribution. Curved arrow shows the direction of particle movement. In this case the position of laser spot is at the maximum of interface bending.

For relatively thick dyed liquid layers (well above 20 μm) after launching intense laser illumination (I ~ 200 W/cm^2^, λ = 405 nm) placed at a distance of 300 to 500 μm from the interface it begins to bend and next abruptly jumps toward the beam center and then it is locked at this place (*x*_*c*_*, y*_*c*_). This phenomenon has been registered and shown in SM1 movie in Supplementary Material, using luminescent particles embedded in solution in order to trace the movement of streams of fluid. When this happens, no further bending is possible as the maximum temperature at the surface has been reached for a given laser power. The self-amplification process is finished and the whirl becomes extremely stable (e.g. can last several minutes). The example of a stable whirl in pNA-dioxane solution excited by Gaussian laser beam was photographed by us and presented in Fig. 3 but its dynamics is shown in SM1. In fact, we composed Fig. 3 photograph from the movie from which we took several frames, each frame has been colored, so the particle linear velocities could be evaluated, amounting around 2.4 mm/s for loops of diameter of 30 μm and around 0.6 mm/s for loops of diameter of 2 mm. For the first time, up to our knowledge, the particle trajectories are so precisely determined allowing for a detailed numerical modelling, using the COMSOL platform, of the described phenomenon.

We call the whole described above phenomenon a *light-induced two-dimensional Marangoni whirl formation* to underline a difference from the 3D Marangoni effect, e.g. the one resulting in formation of Bénard cells^37^.

### Liquid-air interface bending dependence on external parameters

**Figure 4:**
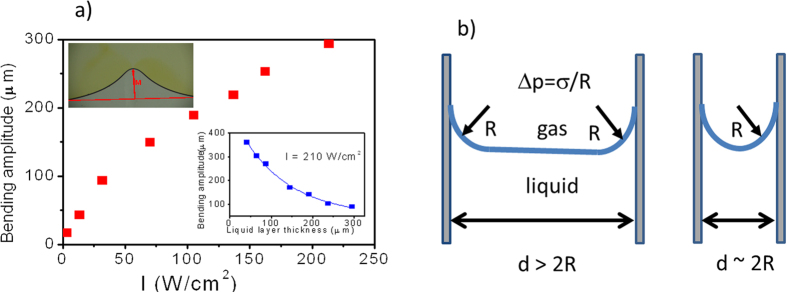
Laser-induced interface distortion. (**a**) Amplitude of liquid-air interface bending in function of laser beam intensity (layer thickness *d* = 70 μm, λ = 405 nm). Inset: dependence of amplitude of liquid-air interface bending on thickness of liquid layer confined between two glass plates. Line shows the elasticity limit of the interface above which it abruptly returns to the initial position. (**b**) Schematic side-view of liquid layer wetting glass surfaces with dependent on thickness strength of capillary forces.

Laser beam positioned at a distance of 0.5 mm from the gas-liquid interface inside the absorbing liquid promotes the interface bending only if the temperature increase at this border is large enough to substantially decrease the surface tension coefficient. Then a stream powered by Marangoni effect of warm liquid is formed and moving from the layer bulk toward the closest to the beam position place at the interface. Then the stream splits symmetrically to the left and right sides of the flat surface (cf. Figs 2 and 3). However, this situation is unstable. Appearance of the bending in response of heating the liquid by laser point source gives additional self-amplification to the thermocapillary Marangoni effect by reducing the flow constraints present at a flat surface. The momentum of moving liquid mass must not change in the central point by ±90° with respect to the incoming from a bulk liquid stream direction (as it happens for flat interface) but at some smaller angle. This happens when all heat dissipation channels equilibrate heat supply, e.g. either by heat conduction to the substrate, solvent evaporation process, temperature equilibration by liquid mixing, etc. Bending stops to increase when an interface is reaching the beam center. The amplitude of interface bending is shown in Fig. 3 by a vertical arrow and in inset to Fig. 4. Bending amplitude can formally be defined as the difference in position of maximum of interface when laser is on with respect to the reference interface position when laser is off. We performed two experiments in order to establish the dependence of amplitude of interface bending on external parameters, like laser power and liquid layer thickness *d* confined between two glass walls. Sample was horizontally positioned under optical microscope and illuminated from the bottom by slightly focused laser beam (spot diameter ~200 μm). We started the laser and placed the beam center exactly at the initially flat interface. In this case no bending is observed. Then we slowly shifted the laser beam toward liquid phase bulk until maximum achievable bending has been obtained, i.e. until the escape of interface from the laser spot position occurred (cf. also SM2, where the crystallization of the p-NA has been captured at the place outside the liquid phase). In Fig. 4a we show the experimental dependence of maximum interface bending amplitude with respect to the flat datum in function of laser intensity (from 3 up to 210 W/cm^2^) for a liquid layer of thickness *d* = 70 μm. On increasing the laser beam intensity the sublinear increase of maximum bending amplitude has been measured (cf. Fig. 4a). Under 210 W/cm^2^ irradiation of 70 μm layer a bending of 300 μm has been reached. However, we have found that bending amplitude is a strong function of layer thickness. Therefore, in another experiment, using the same concentration of a dye in a solvent and constant laser intensity of 210 W/cm^2^ we measured the bending amplitude in function of liquid layer thickness controlled by placing the suitable spacers between glass plates. Results presented in inset to Fig. 4a evidence that the highest bending of interface has been achieved for the thinnest layer. This was an unexpected result as the amount of absorbed radiation energy decreases with decreasing thickness, as well as the heat production. We concluded that for thin layers the capillary effects are responsible for the much easier deformation of the interface by laser beam.

The mechanism of this enhancement can be understood as follow. The surface tension between the liquid and air (described by coefficient *σ*_*LA*_) is usually greater than its surface tension (adhesion) with the solid glass walls of a container (described by coefficient *γ*_*LS*_), i.e. liquid is wetting the substrate. Both coefficients *σ*_*LA*_ and *γ*_*LS*_ are temperature dependent, but the role of adhesion represented by γ_*LS*_ rises on decreasing the layer thickness *d*. Marangoni flows occur in bulk of a liquid i.e. in plane of glass walls and for thin layer light energy is more efficiently used to move smaller volume of fluid than for a thick one, besides the heat escape by evaporation is depressed due to free surface decrease with diminishing thickness (cf. Fig. 4b). From our experiments for dioxane-pNA system it is clear that thermocapillary effects enhance the efficiency of light-induced interface bending.

### Formation and transport of gas bubbles

**Figure 5:**
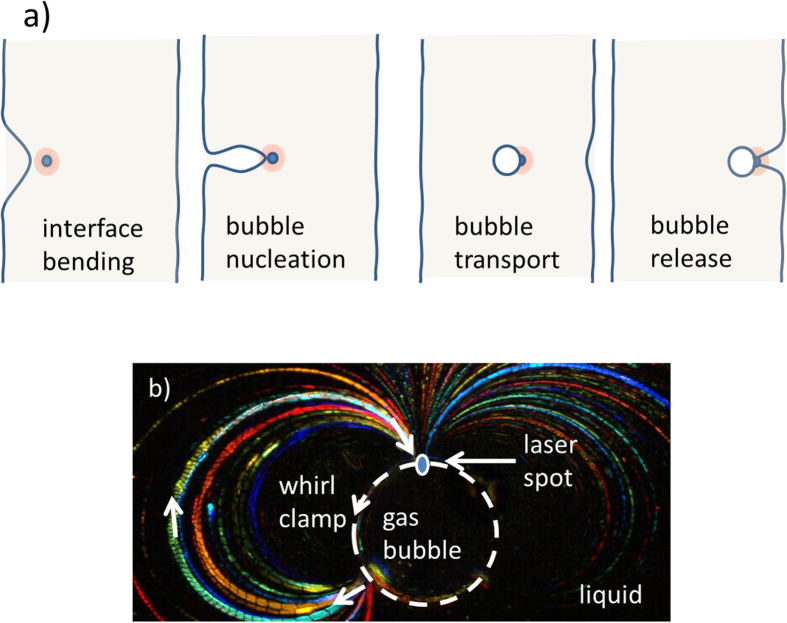
Optical tweezers for a gas bubble. (**a**) Schematic view of transport of a gas bubble through liquid layer using laser beam and optical whirl formation via Marangoni effect (cf. SM3). (**b**) Photograph of a cylindrical gas bubble clamped by fluid streams viewed in dark field where trajectories of particles are visualized.

For relatively thin dioxane-pNA layers (below 20 μm) an interesting phenomenon can occur (cf. Fig. 5). Upon a slow movement of the laser beam position that is dragging interface the distance from the flat datum becomes so large that the shape of interface elongates enormously leading finally to separation of the air bubble by closing the interface edges together at the area shown in Fig. 5a (second diagram). Immediately the bubble attains the cylindrical shape. The cylindrical bubble is pinned up (trapped) to the laser beam (cf. Fig. 5a third diagram) and can be transported together with the movement of the beam what clearly illustrates the movie SM3 attached to the supplementary material. In Fig. 5b using the described earlier technique we photographed the whirls of fluid accompanying the gas bubble. The liquid flow pattern consists of a strong main flow directed towards the position of laser spot on the edge of the bubble and two symmetric rotation flows on either side of it. The velocity of the flow is of the order of mm/s. Currently, we cannot determine precisely the temperature distribution around this cylindrical bubble and its difference between laser spot position and the point where the flow detaches from the liquid-gas interface.

The whirls effectively clamp the bubble when the beam center is positioned exactly at the edge of the liquid-gas interface. Described here mechanism is different from few other mechanisms reported in literature so far, namely trapping by radiation forces^38^, trapping by two-dimensional interference pattern^39^, by photothermal effect with the beam focal point in the bubble center^40^ or using thermoplasmonic Marangoni effect^26^. Having such optical trapping mechanism, one is able to move freely the gas bubble pinned-up to the laser beam through the layer until the other side of the liquid is reached. The process can end with a release of a gas bubble at the other side of liquid. In this fashion, a transport of a pico-liter amount of gas through liquid membranes becomes feasible. The process can be repeated several times both ways. Having two different gases on both sides of liquid membrane and using two laser beams guided separately by systems of mirrors one can perform reactions of two gases under microscope that could be beneficial in many nanotechnological processes and may help in controlled synthesis of various single nanoparticles. The thermocapillary forces used for transport of bubbles were estimated basing on the method described by Ivanowa *et al*.^21^ where acceleration toward laser spot and size of a bubble have to be measured. Observations of bubble movement and acceleration in the vicinity of laser spot gave us similar values as those reported earlier, i.e. exceeding 10 nN, these values have been obtained for experiments with 70 μm thick layers.

### Surface tension driven “micro-scale catapult”

**Figure 6:**
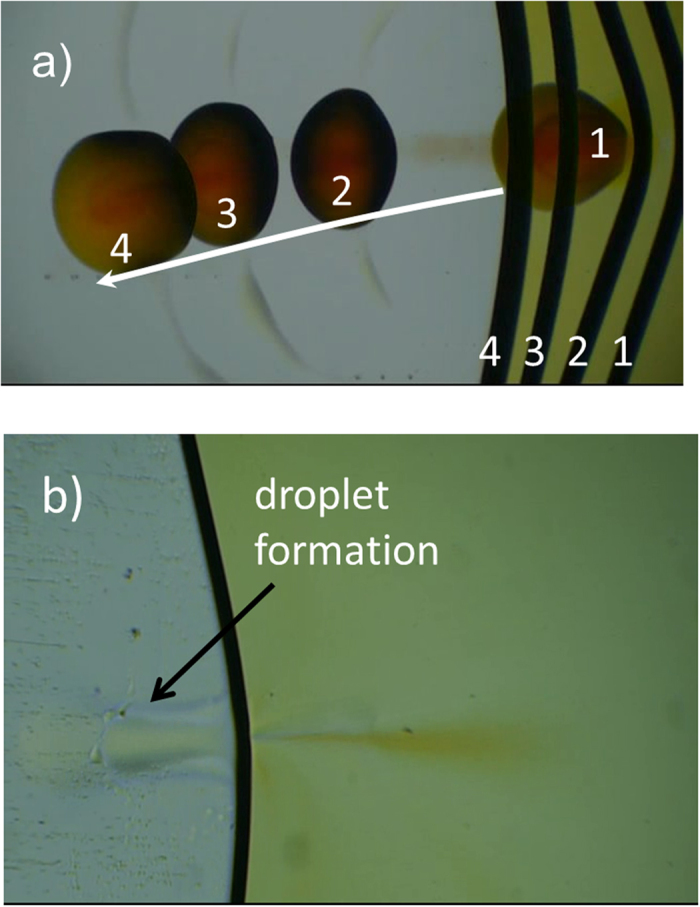
Optically driven droplet “micro-catapult”. (**a**) Sequence of four photographs during the process of formation of sessile droplet of dense p-NA solution with laser light “on” and its subsequent ejection by relaxation of deformed interface with laser light “off” (cf. also SM4). Numbers show the mutual positions of interface and droplet in function of time. (**b**) Detailed photograph showing stream of liquid ejected via interface liquid-gas and starting formation of a droplet over glass plate (see also SM5).

We also demonstrate another spectacular phenomenon using the Marangoni effect in two-dimensions, namely a formation of a “micro-scale catapult”. Using the elastic energy stored by liquid-air interface bending induced by laser light we show how microscopic size droplets (here droplets of concentrated p-nitroaniline in 1,4-dioxane) can be pushed away from the interface which is shown in Fig. 6 and SM4 movie. Droplets production and their subsequent ejections can be realized in a very repeatable way. Understanding and experimental realization of this phenomenon was quite challenging. As mentioned above, laser light-induced Marangoni effect can lead to interface bending. For a beam center being set in the bulk of the layer several tens of micrometers far from the interface a whirl of colored solution containing p-nitroaniline is formed. The initially broad stream of liquid becomes limited to a very narrow one (a funnel-like) close to the liquid-gas interface reaching it at a relatively high speed. As mentioned earlier, at the interface, this stream is split into two equal parts that change their momenta by almost 90 degrees and next are moving in almost opposite directions close to the interface.

Such a dramatic flux splitting is difficult. Interface is not a solid barrier for a liquid stream hitting it normal to the interface and a part of liquid evidently cross the interface and move in the form of a very smooth thin layer stream wetting the glass outside the interface. Surprisingly the evanescent outside stream is enriched by molecules or clusters of pNA because we observe a formation of a very colored dense sessile droplet fast growing in the front of the stream. Despite that the droplet is thinner than the distance between container walls (70 μm) it shows absorption characteristic of dense p-nitroaniline solution that was measured spectroscopically. The absorption edge in 1,4-dioxane- p-nitroaniline is rising rapidly below 420 nm. The fluid density of the droplet is higher than the density of 1,4-dioxane (0.995 g/cm^3^) but lower than the density of p-NA (1.333 g/cm^3^). The concentrated solution of p-nitroaniline shows a surface tension larger than that of pure 1,4-dioxane as it is dependent on composition approaching *σ*_p-NA_ = 60.3 mN/m. We frequently observed the growth of p-NA crystals from the droplet when evaporation of solvent has occurred. The stable droplet formation is possible because it is rotating around the z-axis, the direction of this rotation is random enabling exchange of stream of fluid momentum into droplet rotation. When a droplet has reached a suitable size, we cut-off a laser beam to cease a Marangoni effect. Subsequently the temperature gradients responsible for an interface bending vanished and it has returned to its initial (not bended) position. The movement of relaxed interface pushes vigorously the thin 1,4-dioxane layer covering the bottom glass and also hits the dense droplet that moves away from its initial position. A “micro-catapult” for droplets is formed evidencing the exchange of light power into mechanical work of droplet acceleration. Droplets flow away over wetted glass surface (cf. SM4 and Fig. 6) loosing with a distance its circular shape and height and finally spread out and disappear. In Fig. 6a superposition of few photographs illustrating the process of droplet ejection is shown. Opening the laser source again repeats the whole process of droplet formation in front of jet of fluid stream and subsequent ejection due to elastic energy stored in bend interface takes place on laser light cut-off.

## Conclusions

The phenomena demonstrated in this work are quite general as they describe light-matter interaction via process of light absorption. This is the most efficient way of photons energy transfer into mechanical work. At the same time, the described phenomena (e.g. bubble transport using laser spot and spectacular droplet formation and ejection due to Marangoni effect) are very complex, as their description requires solving of coupled differential equations of heat transport, mass transport, hydrodynamics, surface phenomena and light absorption. The system that we are using 1,4-dioxane with pNA is quite interesting and unique. In our previous reports^33^,^34^ we described the whirl formation in 3D droplets. The phenomena described in those reports require 3D modelling of fluid mechanics by solving the Navier-Stokes equation^36^ with complicated boundary conditions. In this work, we prepared experiments that confine the Marangoni phenomenon to 2D, i.e. to the thin liquid layer with boundary interface. This enabled direct observation of paths of fluid flow due to optically induced thermocapillary Marangoni effect under optical microscope. The visualization technique that we employed uses tracing with CCD camera of small particles embedded in liquid in the white field. Alternatively, we used luminescent particles and/or polarized illumination under crossed polarizers and observed the phenomenon in the dark field. The spectacular example of complex fluid flow visualization is shown in SM6 movie where an isotropic phase of liquid crystal was used. All these techniques unambiguously confirm basic phenomena underlying the optical Marangoni effect. The proposed optical trap mechanism allows for broad interval of cylindrical bubble diameters and large range of distance for bubble displacements (of few mm). Using laser thermocapillary traps, it is possible to develop methods for manipulating multiple bubbles using spatial light modulators and performing small size syntheses, particle movements, and their deposition on a surface and possibly apply in biomedical investigations^40^,^41^,^42^.

## Additional Information

**How to cite this article**: Miniewicz, A. *et al*. Marangoni effect visualized in two-dimensions Optical tweezers for gas bubbles. *Sci. Rep.* **6**, 34787; doi: 10.1038/srep34787 (2016).

## Supplementary Material

Supplementary Information

Supplementary Video

Supplementary Video

Supplementary Video

Supplementary Video

Supplementary Video

Supplementary Video
