# Supplementary material for: Marangoni effect visualized in two-dimensions Optical tweezers for gas bubbles
Source: Sci Rep. 2016 Oct 7;6:34787. doi: 10.1038/srep34787 (PMC5054428; doi:10.1038/srep34787)
Supplement: Supplementary Information [file srep34787-s1.pdf]

SUPPLEMENTARY INFORMATION

Marangoni effect visualized in two-dimensions

Optical tweezers for gas bubbles

A. Miniewicz, S. Bartkiewicz, H. Orlikowska, K. Dradrach
Advanced Materials Engineering and Modelling Group

Faculty of Chemistry, Wroclaw University of Science and Technology

50-370 Wroclaw, Poland

As an illustration to the two-dimensional thermocapillary Marangoni effect we present few examples in the form of movies taken under an optical microscope. In each movie (except the last one) we have study the layer of p-NA:1,4-dioxane solution in between two glass plates separated by the distance of the tens of micrometers. The microscope objective has magnification 10x, and white as well as dark-field images has been photographed by a conventional CCD camera. Laser used for the generation of Marangoni effect was a cw semiconductor diode of power 70 mW working at 405 nm wavelength. Viewing field was about 2x2 mm^2.

SM1: Creation of air-liquid interface bending by laser beam absorbed in p-NA-1:4-dioxane solution. The movie is taken under crossed-polarizers under microscope. Laser spot (seen at the upper part of a movie) on approaching to the initially flat interface (diffused line at the bottom) causes its bending. Fluorescent in green particles mark the liquid flow toward the surface. Their movement becomes more vigorous when laser spot approaches the interface. Then at certain position interface is bending so much that it jumps to the beam spot position. The stable whirl of liquid is formed.

SM2: In this movie taken in white field the air-liquid interface of p-NA-1:4-dioxane solution (the black line) is monitored in function of changing laser light position situated initially at the interface. Bending of interface is gradually increasing. At some moment one can observe the formation of a Sessile droplet of dense p-NA-1:4-dioxane solution outside the liquid phase. This process leads finally to p-NA crystallization.

SM3: The movie illustrates how in a thin (20 μm) layer of the studied solution laser beam is able to trap the cylindrical gas bubble and transport it from one to another side of the liquid layer. The gas bubble is drag by the whirls of liquid around the bubble accompanying the position of laser

spot situated at the edge of the bubble. Here these whirls are invisible but using another technique we visualized them and shown in SM4.

SM4: A microscopic “catapult” for the Sessile droplets due to relaxation of energy stored in the air-liquid interface bending due to Marangoni effect. Laser spot, here invisible, is situated to the right side of the movie. Its presence bends the interface to the right and a dense p-NA:1,4-dioxane solution forms a droplet on the glass slide. When the droplet is formed the laser light is switched-off and the interface returns to its initial position pushing the droplet to the left. The process could be repeated several times until the formation of the droplets is possible.

SM5: The movie illustrates the formation of Marangoni flows induced by laser beam and an accompanying phenomenon of ejection of some stream of liquid through an interface toward a gas phase and formation of sessile droplet.

SM6: An example of laser light-induced Marangoni flows in two-phase system. A liquid crystal is dragged by the Marangoni flow toward the interface at which the laser spot is situated. The visualization of the fluid flows is possible due to isotropic-to-nematic phase transition in liquid crystal driven by local temperature field.
